# The number of CD34+CD38+CD117+HLA-DR+CD13+CD33+ cells indicates post-chemotherapy hematopoietic recovery in patients with acute myeloid leukemia

**DOI:** 10.1371/journal.pone.0180624

**Published:** 2017-07-05

**Authors:** Runxia Gu, Hui Wei, Ying Wang, Dong Lin, Bingcheng Liu, Chunlin Zhou, Kaiqi Liu, Benfa Gong, Shuning Wei, Guangji Zhang, Xiaoyuan Gong, Yuntao Liu, Yan Li, Xingli Zhao, Shaowei Qiu, Huijun Wang, Min Wang, Yingchang Mi, Jianxiang Wang

**Affiliations:** 1 Department of Leukemia, Institute of Hematology, Hospital of Blood Diseases, Chinese Academy of Medical Sciences, Tianjin, China; 2 Department of Pathology and Lab Medicine, Institute of Hematology, Hospital of Blood Diseases, Chinese Academy of Medical Sciences, Tianjin, China; 3 State Key Laboratory of Experimental Hematology, Institute of Hematology and Blood Diseases Hospital, Chinese Academy of Medical Sciences & Peking Union Medical College, Tianjin, China; Sun Yat-sen University, CHINA

## Abstract

Hematopoietic recovery is considered to be associated with the number of multipotent hematopoietic stem cells in the bone marrow, as observed in functional assays involving stem cell transplantation. However, there is little evidence related to hematopoietic recovery in non-transplantation settings, which is accomplished by endogenous hematopoietic cells. A recent study suggested that progenitors are the main contributors during this steady-state hematopoiesis, which differs from exogenous transplantation. We hypothesized that endogenous progenitor support post-chemotherapy hematopoietic recovery. To investigate the potential impact of these progenitor cell percentage on hematopoietic recovery, we retrospectively analyzed the percentage of CD34+CD38+CD117+HLA-DR+CD13+CD33+ cells (P cells) and hematopoietic recovery in 223 newly diagnosed acute myeloid leukemia patients during two courses of consolidation chemotherapy after complete remission. We found that a lower P cell percentage was significantly associated with prolonged neutropenia recovery time after the first and second courses of consolidation chemotherapy (p = 0.001; p = 0.045, respectively). We also observed similar results with regard to platelet recovery time after the first course of consolidation chemotherapy (p = 0.000). Univariate analysis showed that P cell percentage and consolidation chemotherapy regimens, and not gender, age, induction chemotherapy regimens, infection grade, WHO classification and NCCN risk category, were associated with neutrophil recovery after chemotherapy. Multivariate analysis demonstrated that P cell percentage is an independent factor affecting neutrophil recovery capacity for both the first and second courses (p = 0.008; p = 0.032, respectively). Our results indicate that CD34+CD38+CD117+HLA-DR+CD13+CD33+ cells before each course of chemotherapy is independently associated with chemotherapy-related hematopoietic reconstitution capacity. These findings may help modify future chemotherapy regimens based on progenitor cell percentages.

## Introduction

Hematopoietic recovery is one of the key factors affecting the outcome of chemotherapy. Faster bone marrow recovery leads to fewer adverse consequences and enables patients to proceed with further courses of chemotherapy without delay. A delayed bone marrow recovery can lead to an uncontrolled infection that results in treatment failure. The hematopoietic toxicity of chemotherapy is an important factor in determining the doses for treatment regimens. Unfortunately, there are no adequate models to predict toxicity from chemotherapy, and little progress has been achieved in this area.

Abundant evidence has shown that hematopoietic stem cells are responsible for the regeneration of blood cells[1–3]. Evidence supporting this view has been largely acquired through the use of functional assays involving transplantation[4–5]. The number of CD34+ hematopoietic stem cells from a donor is associated with a recipient’s hematopoietic recovery after bone marrow transplantation[6]. However, there is no direct evidence to show what factors are responsible for hematopoietic recovery after chemotherapy.

Recent evidence suggests that a large number of long-lived “short-term” stem cells, called progenitors, are the main factors in steady-state hematopoiesis during most of adulthood rather than the classically defined hematopoietic stem cells [7–8]. In this paper, we chose a group of cells (P cells) with the phenotype CD34+CD38+CD117+HLA-DR+CD13+CD33+. We investigated the relationship between the number of these cells and hematopoietic recovery after chemotherapy. We found that the percentage of these cells in the bone marrow had a positive correlation with hematopoietic recovery. According to our multivariate analysis, the percentage of these P cells is the independent factor related to the time of neutropenia after chemotherapy.

## Methods

### Patients

In this study, we retrospectively analyzed 223 patients with de novo acute myeloid leukemia (AML) at the Institute of Hematology, Hospital of Blood Diseases, Chinese Academy of Medical Sciences from May 2012 to January 2016.

All patients recruited in our study were from a previously registered prospective randomized clinical trial launched by our department: “A Phase III Study on optimizing treatment based on risk stratification for acute myeloid leukemia, Registry Number: ChiCTR-TRC-10001202; Date of Registration: 2010-12-02; Study ID: 201002024; AML2010-01”.

This clinical trial lasted for 5 years and ended in 2016. In this clinical trial, patients newly diagnosed as AML were randomly grouped into two groups receiving either standard HAD induction chemotherapy (HAD-standard, Ara-C 100mg/m^2^/d d1-7), or intermediate HAD regimen (HAD-intermediate, Ara-C 100mg/m^2^/d d1-4, 1g/m^2^/q12h d5-7). Then, patients with complete remission after induction chemotherapy were further randomized to receive two types of consolidation therapy (high-dose cytarabine, Ara-C 3g/m^2^/q12h d1-3 (HDAC) vs. intermediate-dose cytarabine combined with anthracycline, Ara-C 1.5g/m^2^/q12h d1-3 (IDAC)). Regarding this retrospective study, patients from the registered clinical trial who maintained complete remission (CR) after both induction and two courses of consolidation therapy were included. Patients with acute promyelocytic leukemia or leukemia either transformed from myelodysplastic syndrome or secondary to other malignancies were excluded in this research. We reviewed the data from bone marrow flowcytometry before each course of consolidation therapy, in which the CD34+CD38+CD117+HLA-DR+CD13+CD33+ progenitor cell percentage in the bone marrow was analyzed (the P cell percentage has been available since May 2012). Platelet recovery time and time of neutropenia were counted for the evaluation of hematopoietic recovery ability after chemotherapy.

The authors had access to information that could identify individual participants during or after data collection. The definition of neutropenia time was the number of days during which the absolute neutrophil count (ANC) was less than 0.5×10^9^cells/L. The delayed time to ANC recovery was defined as greater than one SD above the mean of neutropenia time. The platelet recovery time (PLT recovery time) was defined as the number of days from the day the platelet count was <20×10^9^ cells/L to the day restored to 20×10^9^ cells/L, and free of platelet infusion for at least one week. Granulocyte-colony stimulating factor (G-CSF) was applied at 5μg/kg/d during the time of neutropenia.

This research was approved by the ethical committee in the Institute of Hematology and Blood Diseases Hospital and all procedures were in accordance with the Helsinki Declaration of 1975, as revised in 2008. All participants provided written informed consent. There were no other treatments administered, prescribed, or modified as part of this study.

### Statistical analysis

Comparisons of P cell percentage, the time of neutropenia and the platelet recovery time before and after each consolidation chemotherapy were analyzed by Wilcoxon non-parametric test. The Mann-Whitney non-parametric test was used for the analysis of two independent groups. The Chi-square test was used for categorical data. All p-values represented were two-sided and considered statistically significant when p<0.05. Both univariate and multivariable Cox regression analyses were applied to determine whether the P cell percentage contributes to the time to ANC recovery after each course of chemotherapy. Patients whose neutrophil or platelet count number did not recover at the end of the course were censored. Multivariate Cox analysis was performed (backwald, wald), including all variables from univariate analysis with a p value <0.1, Statistical analyses were performed with SPSS 19.0 software.

## Results

### CD34+CD38+CD117+HLA-DR+CD13+CD33+ cell percentages and their changes during consolidation therapy

**Fig 1:**
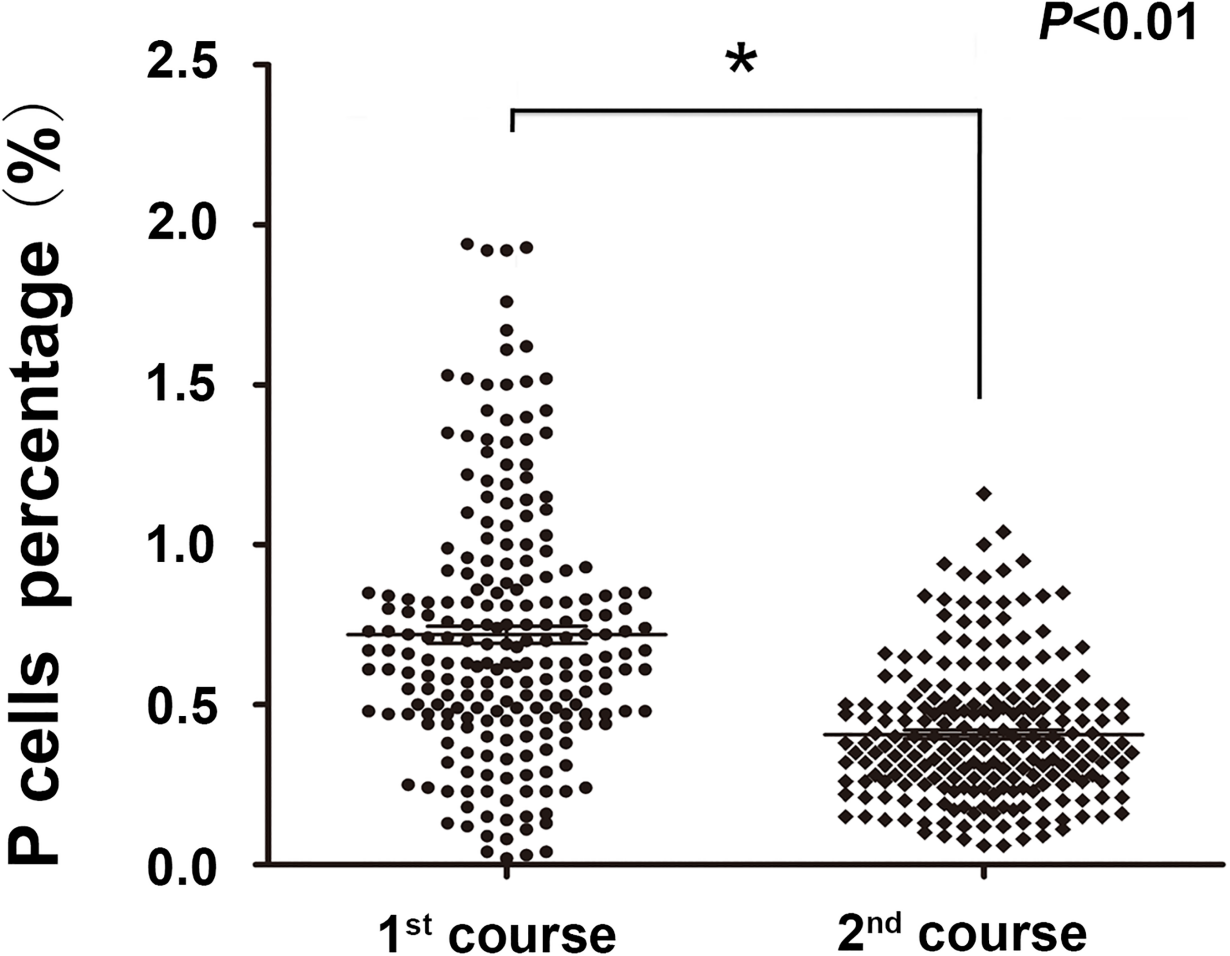
P cell frequency declined with increasing rounds of chemotherapy. The percentage of P cells was evaluated in patients with AML before the first and second course of consolidation chemotherapy by flowcytometry. Each symbol represents a single patient, and the data are shown as the means±SEM of the indicated number of patients.1^st^ course: the first course of consolidation chemotherapy; 2^nd^ course: the second course of consolidation chemotherapy.

In total, 223 *de novo* AML patients were given consolidation chemotherapy after achieving CR. The P cell percentage was determined by flow cytometry before each course of consolidation chemotherapy. As expected, the P cell frequency declined with increasing rounds of chemotherapy. As shown in Fig 1, the median P cell percentages were 0.72% (ranging from 0.02% to 1.94%) and 0.40% (ranging from 0.06% to 1.16%) before the first and second course of consolidation chemotherapy, respectively. The P cell percentage before the second course of consolidation chemotherapy decreased significantly compared with the percentage before the first course (p = 0.000).

### Hematopoietic recovery after chemotherapy is correlated with P cell percentage

**Fig 2:**
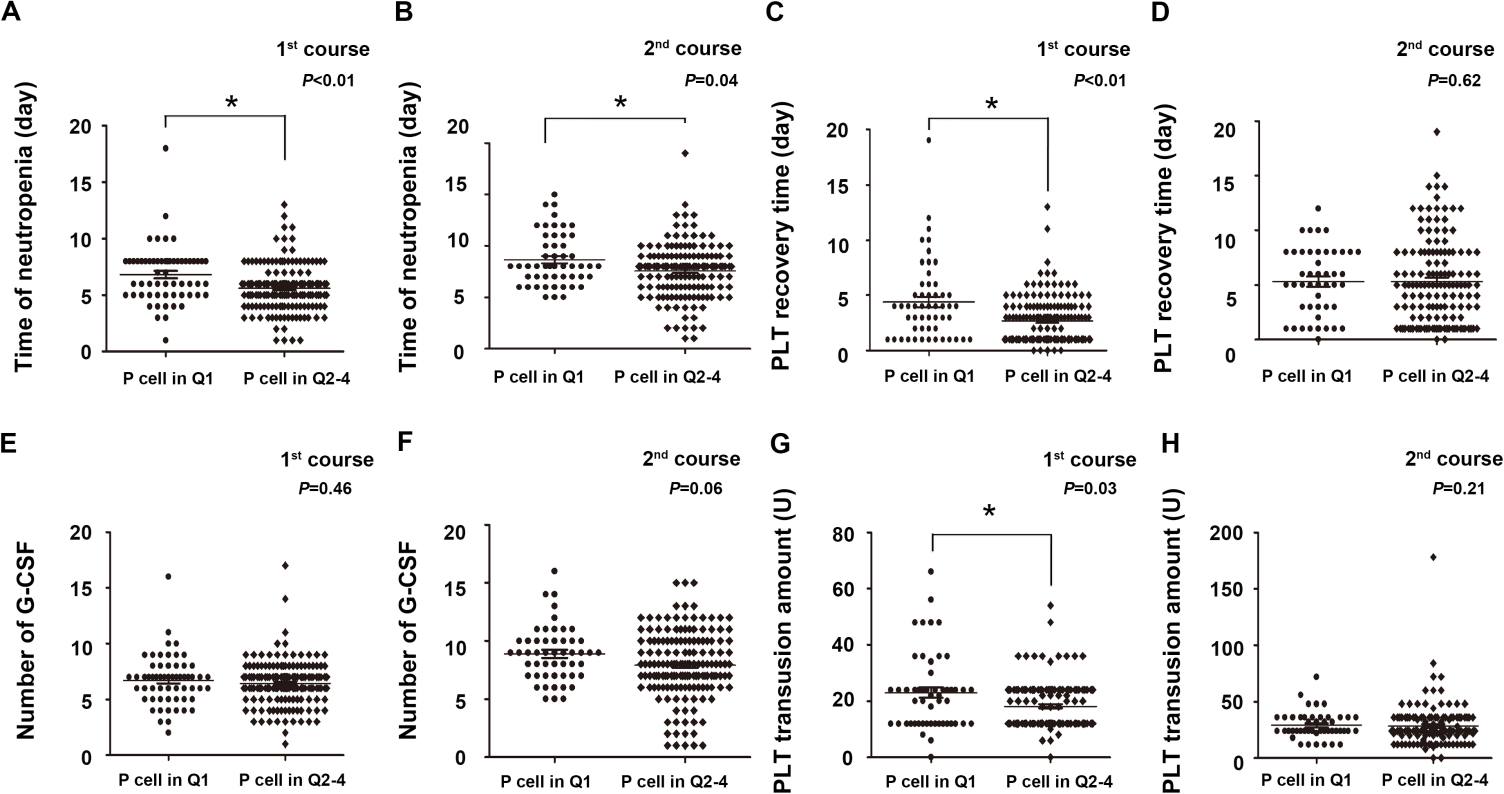
Patients with higher P cell percentages possess better hematopoietic recovery. Evaluation of hematopoietic recovery in patients with different pre-chemotherapy P cell percentages (Q1groupor Q2-4 group) after consolidation chemotherapy. Times of neutropenia after the first (A) and the second (B) courses of chemotherapy were evaluated, as well as platelet (PLT) recovery time (C and D), G-CSF levels (E andF) and platelet transfusion amount (G and H). The data are shown as the means±SEM of the indicated number of patients.

To examine the relationship between the number of P cells and hematopoietic recovery capability after chemotherapy, we combined and ranked P cell frequency into quartiles for the 223 samples, with the lowest P cell frequency in quartile one (Q1) and the greatest in Q4. Patients with a lower P cell frequency were compared with the remaining patients for differences induration of neutropenia after chemotherapy. Patients whose neutrophil or PLT count number did not recover at the end of the course were excluded. Patients in Q1 had a significantly longer mean time of neutropenia than those in Q2 to Q4 (Q2-4) (consolidation one, 6.8 days vs. 5.6 days, p = 0.001; consolidation two, 8.7 days vs.7.6 days, p = 0.045; Fig 2A and 2B). Similarly, the platelet recovery time after the first course of consolidation chemotherapy in the Q1group was significantly longer than that in the Q2-4 groups (4.4 days vs. 2.7 days, p = 0.000, Fig 2C). After the second course of consolidation chemotherapy, there was no significant difference in the platelet recovery time between Q1and Q2-4 groups (5.3 days vs. 5.3 days, p = 0.619, Fig 2D).

Because G-CSF is applied to accelerate hematopoietic recovery in clinical practice, the cumulative amount of G-CSF used per kg of body weight was analyzed to exclude the possibility that the difference in hematopoietic recovery capability was caused by G-CSF. We found that Q1 group patients also need more G-CSF amount after the first and second course of chemotherapy, though statistical analysis showed no significant difference (consolidation one, 6.7 vials vs. 6.4 vials, p = 0.458; consolidation two, 8.9 vials vs. 7.9 vials, p = 0.056, Fig 2E and 2F). The findings on the usage of G-CSF indicate that the difference in hematopoietic recovery capability was not caused by G-CSF. Instead, these results provide further evidence that patients with fewer P cells have a weak hematopoietic recovery capability because they received more G-CSF. In addition, we analyzed the amount of platelet transfused in these two course of chemotherapy. We found that the amount of platelet transfused in the Q1 group was significantly more than the amount transfused in the Q2-4 groups (23U vs. 18U, p = 0.034) after the first course of chemotherapy (Fig 2G). But there was no significant difference after the second course of chemotherapy (29U vs. 28U, p = 0.206, Fig 2H). Taken together, these findings further strengthened the fact that patients with fewer P cells have a weak hematopoietic recovery capability and thus requires more G-CSF and platelet transfusion.

### P cell percentage is an independent factor affecting hematopoietic recovery after chemotherapy

The relevant clinical characteristics of the 223 patients, such as gender, consolidation chemotherapy regimens, World Health Organization (WHO) classification and National Comprehensive Cancer Network (NCCN) risk categories, were equivalent between patients in Q1and Q2-4 in both consolidations one and two, with the exception of the median age in years being higher in Q1 than in Q2-4 for consolidation one (p = 0.000, Table 1). And patients who received HAD-intermediate regimen in Q1 group outnumbered Q2-4 group in consolidations one (p = 0.040, Table 1). Cox regression confirmed that the P cell percentage was predictive of neutropenia recovery time in both the first and second courses of consolidation chemotherapy by univariate analysis (consolidation one, p = 0.010; consolidation two, p = 0.042, Table 2). Univariate analysis showed that consolidation chemotherapy regimens (HDAC vs. IDAC combination) were also associated with neutropenia recovery capability in the second course of consolidation (p = 0.045, Table 2). Other factors, such as gender, age, induction chemotherapy regimens (HAD-standard vs. HAD-intermediate), infection grade (grade1-2 vs. grade 3–5), WHO classification and risk category (unfavorable vs. intermediate vs. favorable), were not correlated with ANC recovery capability. Then P cell percentage, consolidation chemotherapy regimens and infection grade were taken into multivariable analysis, and the P cell percentage remained independently predictive of time to ANC recovery in consolidation one (p = 0.008, Table 3) and two (p = 0.032, Table 4), so did chemotherapy regimens in consolidation two (p = 0.034, Table 4).

**Table 1:** Clinical characteristics of the 223 patients with AML.

	Consolidation one			Consolidation two		
	Q1 Patients (n = 61)	Q2-4 Patients (n = 162)	P	Q1 Patients (n = 56)	Q2-4 Patients (n = 167)	P
**Age(years)**						
Median	41	33	**0.000**	36	35	**0.537**
Range	16–54	15–53		15–52	15–54	
**Gender**						
Male	31(50.8%)	94(58.0%)	**0.334**	28(50.0%)	97(58.1%)	**0.292**
Female	30(49.2%)	68(42.0%)		28(50.0%)	70(41.9%)	
**Induction chemotherapy regimens**						
HAD-standard	26(42.6%)	94(58.0%)	**0.040**	33(58.9%)	87(52.1%)	**0.375**
HAD-intermediate	35(57.4%)	68(42.0%)		23(41.1%)	80(47.9%)	
**Consolidation chemotherapy regimens**						
HDAC^1^	36(59.0%)	73(45.1%)	**0.063**	28(50.0%)	81(48.5%)	**0.846**
IDAC^2^	25(41.0%)	89(54.9%)		28(50.0%)	86(51.5%)	
**NCCN risk categories**^**3**^						
Good	25(43.1%)	71(44.1%)	**0.778**	28(50.0%)	68(41.7%)	**0.207**
Intermediate	26(44.8%)	62(38.5%)		20(35.7%)	68(41.7%)	
Poor	7(12.1%)	28(17.4%)		8(14.3%)	27(16.6%)	
No information	3	1		0	4	
**WHO classification**^**4**^						
AML with t(8;21) (q22;q22)	7(11.5%)	36(22.2%)	**0.276**	12(21.4%)	31(18.6%)	**0.692**
AML with abnormal BM eosinophils and inv(16)(p13;q22) or t(16;16)(p13;q22)	1(1.6%)	4(2.5%)		0(0.0%)	5(3.0%)	
AML with 11q23 abnormalities	2(3.3%)	6(3.7%)		2(3.6%)	6(3.6%)	
Other	51(83.6%)	116(71.6%)		42(75.0%)	125(74.9%)	

1. HDAC, high-dose cytarabine.

2. IDAC combination, intermediate-dose cytarabine combined with anthracycline.

3. NCCN AML risk Categories (2015), the patients with on information in NCCN risk category ware not included in χ2 analysis.

4. WHO AML classification (2016).

**Table 2:** Univariate analysis for ANC recovery.

	Consolidation one			Consolidation two		
	HR	P	95% CI	HR	P	95% CI
P cell percentage	1.494	0.010	1.102–2.027	1.398	0.042	1.013–1.928
Age	1.000	0.992	0.988–1.013	0.998	0.815	0.986–1.011
Gender	0.953	0.731	0.762–1.252	0.826	0.181	0.625–1.093
Induction chemotherapy regimens	0.912	0.503	0.697–1.194	0.866	0.306	0.657–1.141
Consolidation chemotherapy regimens	0.971	0.831	0.743–1.270	0.747	0.045	0.561–0.994
Infection grade	0.885	0.097	0.765–1.023	0.892	0.204	0.747–1.064
NCCN risk categories	0.881	0.168	0.735–1.055	0.981	0.824	0.824–1.165
WHO classification	0.974	0.653	0.870–1.091	0.985	0.789	0.881–1.101

**Table 3:** Multivariate analysis for ANC recovery in consolidation one.

Consolidation one	No.	B	SE	Wald	HR	*P*	95% CI
P cell percentage							
Q1	61				1		
Q2-4	162	0.411	0.156	6.967	1.508	0.008	1.112–2.047

**Table 4:** Multivariate analysis for ANC recovery in consolidation two.

Consolidation two	No.	B	SE	Wald	HR	*P*	95% CI
P cell percentage							
Q1	56				1		
Q2-4	167	0.354	0.165	4.623	1.425	0.032	1.032–1.968
Consolidation chemotherapy regimens							
HDAC	109				1		
IDAC	114	-0.311	0.147	4.516	0.732	0.034	0.550–0.976

### Chemotherapy-impaired hematopoietic recovery ability

**Fig 3:**
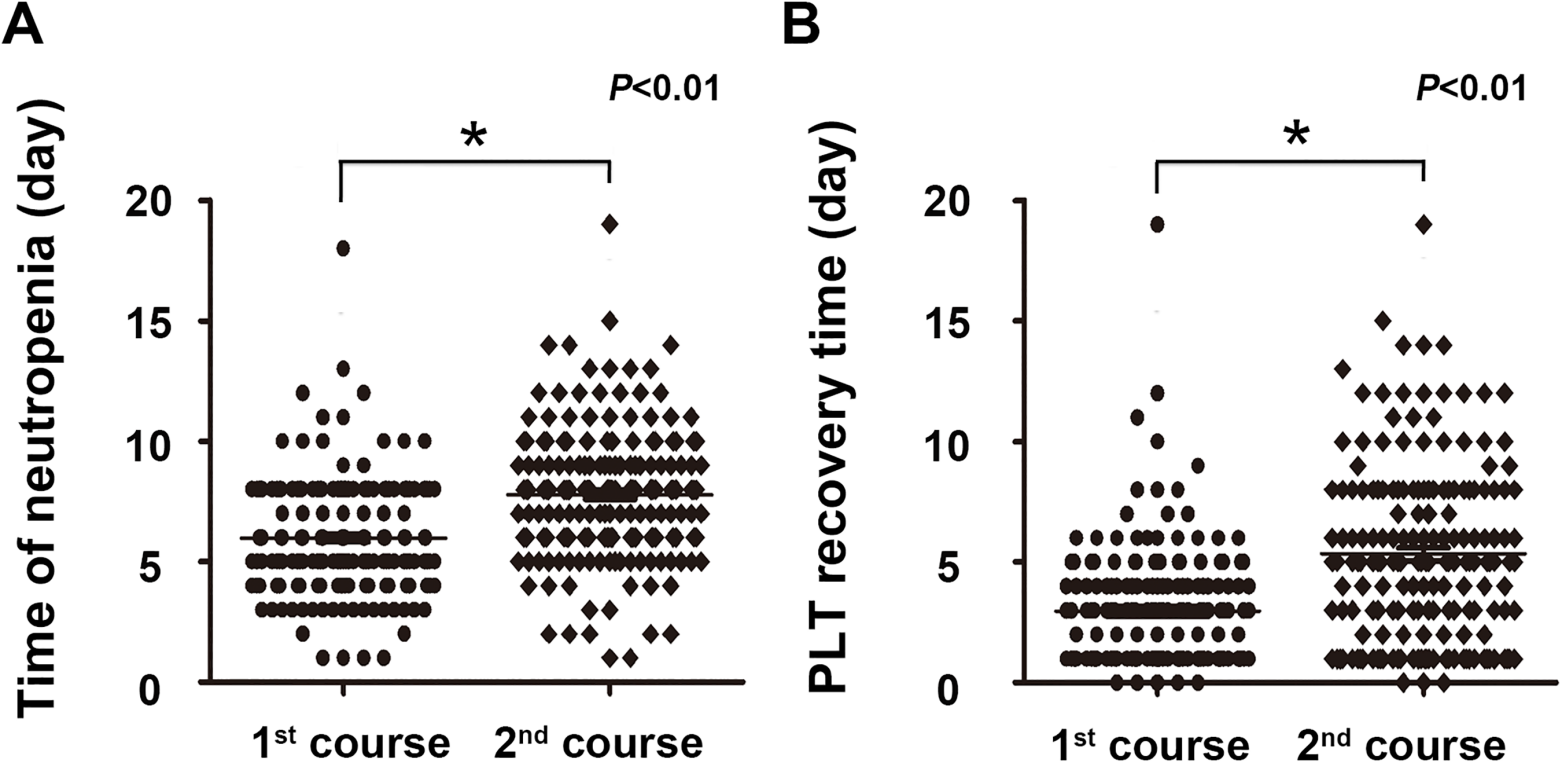
Chemotherapy-impaired hematopoietic recovery ability. Differences in bone marrow recovery capacity represented as time of neutropenia (A) and platelet recovery time (B) after the first and second course of chemotherapy.

It is well known that hematopoietic recovery ability decreases with the continuation of chemotherapy, although there are few references to support this. We found that the P cell percentage decreased with the continuation of chemotherapy (Fig 1), and hematopoietic recovery slowed when there were fewer P cells in the bone marrow (Fig 2A–2D). To explore whether chemotherapy influences hematopoietic recovery capability, we compared the neutropenia time and platelet recovery time between the first and second courses of chemotherapy. Patients whose neutrophil or platelet count number did not recover to normal at the end of any course were excluded. As expected, the time of neutropenia and platelet recovery time after the first course of consolidation chemotherapy were significantly shorter than after the second course (6.0 days vs. 7.8 days, Fig 3A, p = 0.000; 3.0 days vs. 5.3 days, Fig 3B, p = 0.000), This suggests that both the P cell percentage as well as chemotherapy itself are factors affecting hematopoietic recovery. Therefore, we postulated that chemotherapy might impair hematopoietic recovery ability by decreasing the number of P cells.

### P cell percentage is not correlated with infection grade after chemotherapy

Severe neutropenia might lead to an uncontrolled or even lethal infection. Because P cell percentages are inversely correlated with neutrophil recovery after chemotherapy, we compared the rates of microbiologically documented sterile-site infections between patients in Q1and Q2-4. The results revealed that 67.2% (41/61) of Q1 patients experienced Grade 3–5 infections (according to Common terminology criteria for adverse events, CTCAE v4.0) after the first course of consolidation chemotherapy and 83.9% (47/56) of patients after the second course. There were no significant differences in the comparison with the patients in Q2 to Q4 (67.2% (109/162) and 76.0% (127/167), (p = 1.0 and p = 0.21).

## Discussion

We have found that the hematopoietic recovery ability after chemotherapy is associated with the percentage of pre-chemotherapy CD34+CD38+CD117+HLA-DR+CD13+CD33+ progenitor cells (P cells). It is also an independent factor related to time of neutropenia after chemotherapy as shown by multivariate analysis. To our knowledge, this is the first study to show that the number of several types of hematopoietic cells before chemotherapy is related to hematopoietic recovery after chemotherapy.

In clinical practice, some patients show a fast hematopoietic recovery after chemotherapy, while other patients’ hematopoietic recovery is slow. Currently, we do not have direct evidence determining which factors determine the speed of hematopoietic recovery after chemotherapy. We postulated that the difference in recovery time is derived from varied bone marrow reserves or hematopoietic stem cells among different patients [9]. In this study, we found that the percentage of P cells was associated with the time of neutropenia and recovery of platelets and was an independent factor associated with hematopoietic recovery after chemotherapy. To mitigate chemotherapy toxicity, it is potentially possible to use this progenitor cell percentage to modify treatment regimens. We did not find P cell percentages to be correlated with infection grade after chemotherapy. Cytokines, such as G-CSF or GM-CSF, after chemotherapy did not decrease the incidence of fever in younger patients, although they may accelerate WBC recovery [10]. However, cytokine administration after chemotherapy does decrease infectious toxicity and reduces therapy-related mortality and morbidity in elderly patients [11]. Theories based on progenitor cell studies may indicate a more significant role in elderly patients.

In our study, CD34+CD38+CD117+HLA-DR+CD13+CD33+ cells are a group of CD34+CD38+ double-positive cells that represent progenitor cells. This is in accordance with recent studies that “short-term” stem cells, or progenitor cells, support blood cell regeneration [7–8]. Currently, we cannot conclude that P cells are responsible for blood cell regeneration after chemotherapy. Because CD34+CD38- stem cells or other progenitor cells can differentiate into P cells [12], we can only conclude that their presence is an indicator for the potential speed of hematopoietic recovery after chemotherapy. Our study does not rule out the idea that CD34+CD38- stem cells or other progenitor cells are responsible for hematopoietic recovery after chemotherapy, nor are they better predictors of prognosis. Further investigation is needed to clarify these theories in future research.

Stem cell studies are an active topic in current scientific research [13–14]. It is commonly believed that every part of an organism is derived from various stem cells [15–17]. Although it is assumed that the number of stem cells is critical for human life, little direct evidence has demonstrated that the stem cell number is related to human physiological or pathological processes. The only evidence, as we know, is that the number of CD34+ hematopoietic stem cells from the donor is associated with the hematopoietic recovery of the recipient after stem cell transplantation [6]. However, this evidence only proves that the number of exogenous stem cells, not endogenous stem cells, is associated with hematopoietic recovery. Serveries and coworkers’ study [18], together with other epidemiologic studies, suggests a unifying hypothesis that stem cell number is related to breast cancer risk[19].To our knowledge, this is the first study to demonstrate that the number of endogenous stem or progenitor cells is related to human pathophysiological processes. Little is known about the determinants of the number of stem cells in an organism. Guo etal found that the microRNA miR-125a controls the number of hematopoietic stem cells [20]. Currently, we are researching the quality of stem cells. In the future, more research on the quantity of stem cells is necessary.

## Supporting information

S1 File This is the ethics censor-translation. (PDF)

S2 File This is the ethics censor. (PDF)

S3 File This is the certificate for the manuscript editing by AJE. (PDF)

S4 File This is the STROBE_checklist_v4_combined_Plos one. (DOCX)

S5 File This is the original data of all the patients. (XLS)
