# Supplementary material for: The number of CD34+CD38+CD117+HLA-DR+CD13+CD33+ cells indicates post-chemotherapy hematopoietic recovery in patients with acute myeloid leukemia
Source: PLoS One. 2017 Jul 5;12(7):e0180624. doi: 10.1371/journal.pone.0180624 (PMC5498054; doi:10.1371/journal.pone.0180624)
Supplement: S1 File — (PDF) [file pone.0180624.s001.PDF]

Ethics Approved (Translation)

Number	NI2015011-EC-1		
Program	The number of CD34+CD38+CD117+HLA-DR+CD13+CD33+ cells indicates post-chemotherapy hematopoietic recovery in patients with acute myeloid leukemia		
Classification	Scientific study☐ Retrospective analysis■ Medical technology☐ New drug☐ Medical apparatus☐		
Person in charge	Jianxiang Wang		
Review type	Primary review	Review course	Quick review
Files included	Application for retrospective clinical analysis.		

Comments:

This study is approved by our committee.

中国医学科学院血液病医院 伦理委员会

Ethics Committee（seal）:

Date: Dec 26, 2015

Remarks:

If the applicant has any opposed opinion about the decision, a re-evaluation request might be applied with additional materials.

Announcement:

The Ethics Committee comply with Good Clinical Practice of China Food and Drug Adminisration, it also obey the ICH GCP Guideline and the applied laws in China.

Address: 288#, Nanjing Rd., Tianjin, China	Contact: Xiaoxue Zhou	Tel: 022-23909058	E-mail: xyskeyanlunli@126.com
