# Supplementary material for: The number of CD34+CD38+CD117+HLA-DR+CD13+CD33+ cells indicates post-chemotherapy hematopoietic recovery in patients with acute myeloid leukemia
Source: PLoS One. 2017 Jul 5;12(7):e0180624. doi: 10.1371/journal.pone.0180624 (PMC5498054; doi:10.1371/journal.pone.0180624)
Supplement: S2 File — (PDF) [file pone.0180624.s002.PDF]

伦理审查批件

批件号	NI2015011-EC-1		
项目名称	骨髓 CD34+CD38+CD117+HLA－DR＋CD13+CD33+细胞数对急性髓系白血病患者化疗后造血恢复的影响		
项目类别	☐新药试验 ☐器械试剂 ☐横向 IIT ☐科研课题 ■其他		
项目负责人	王建祥		
审查类别	初始审查	审查方式	快速审查
审查文件	回顾性临床研究伦理审批申请书		

审查意见：

同意项目开展

主任委员/副主任委员签字：[illegible]

伦理委员会（盖章）：中国医学科学院血液病医院伦理委员会

日期：2015 年 12 月 26 日

备注：
申请人对审查意见有异议，可向伦理委员会秘书处提交“复审申请”，并提交相应审查资料。

声明：
本伦理委员会的职责、人员组成、运行和记录遵循中华人民共和国食品药品监督管理局颁布的药物临床试验质量管理规范（GCP）和 ICH-GCP 伦理审查原则，并遵守中国的相关法律及法规。

联系地址： 天津市和平区南京路 288 号	联系人：周小雪	联 系 电 话 ：022-23909058	邮箱 xyskeyanlunli@126.com
